# Fingerprick blood samples to measure serum natalizumab concentrations

**DOI:** 10.1177/13524585221136448

**Published:** 2022-11-30

**Authors:** Alyssa A Toorop, Maurice Steenhuis, Floris C Loeff, Suzanne S Weijers, Joep Killestein, Theo Rispens, Zoé LE van Kempen

**Affiliations:** Neurology Outpatient Clinic, Amsterdam UMC location Vrije Universiteit Amsterdam, Amsterdam, The Netherlands; Biologics Laboratory, Sanquin Diagnostic Services, Amsterdam, The Netherlands Department of Immunopathology, Sanquin Research, Amsterdam, The Netherlands; Biologics Laboratory, Sanquin Diagnostic Services, Amsterdam, The Netherlands; Central Diagnostic Laboratory, Amsterdam UMC location Vrije Universiteit Amsterdam, Amsterdam, The Netherlands; Neurology Outpatient Clinic, Amsterdam UMC location Vrije Universiteit Amsterdam, Amsterdam, The Netherlands; Biologics Laboratory, Sanquin Diagnostic Services, Amsterdam, The Netherlands Department of Immunopathology, Sanquin Research, Amsterdam, The Netherlands Landsteiner Laboratory, Academic Medical Centre, University of Amsterdam, Amsterdam, The Netherlands; Neurology Outpatient Clinic, Amsterdam UMC location Vrije Universiteit Amsterdam, Amsterdam, The Netherlands

**Keywords:** Multiple sclerosis, natalizumab, capillary, fingerprick, extended interval dosing

## Abstract

**Background:**

Natalizumab via subcutaneous administration was recently approved for patients with multiple sclerosis.

**Objective:**

In light of personalized extended dosing, in which treatment intervals are prolonged to a concentration cut-off, it would be preferable to measure natalizumab drug concentrations in capillary blood.

**Methods:**

In this cross-sectional study in patients treated with intravenous (IV) natalizumab, capillary blood samples by fingerprick and venous blood samples were collected in 30 participants prior to IV administration of natalizumab.

**Results:**

Natalizumab concentrations were similar with a mean bias of −0.36 μg/mL (95% CI: 1.3 to −2 μg/mL).

**Conclusions:**

This study shows that physicians can monitor natalizumab drug concentrations by a fingerprick, which could be used for personalized extended dosing.

## Introduction

Natalizumab is an effective treatment for patients with relapsing-remitting multiple sclerosis (RRMS). Although approved for administration of 300 mg every 4 weeks, an increasing number of studies show that extended interval dosing of natalizumab beyond 4 weeks can be efficacious, with a lower risk of progressive multifocal leukoencephalopathy (PML) and reduced treatment costs.^1–3^ Natalizumab drug concentrations are lower when applying extended interval dosing compared to standard dosing.^4^ Personalized extended interval dosing, in which treatment intervals are prolonged to a concentration cut-off by monitoring serum natalizumab drug concentrations, is one strategy to ensure adequate receptor occupancy, where a cut-off trough concentration of 1–2 µg/mL is likely adequate.^2,5^

In April 2021, the European Medicines Agency (EMA) approved a subcutaneous (SC) variant of natalizumab.^6^ In patients using natalizumab SC with personalized extended interval dosing, it would be preferable to obtain blood samples without venous access to avoid additional venepunctures. A simple alternative is capillary blood collection using a fingerprick, which can also be performed by patients at home^7^ and enables measurement of natalizumab drug concentrations more frequently. Previous studies have demonstrated that serum concentrations of therapeutic monoclonal antibodies such as adalimumab or infliximab can be evaluated using capillary blood draws instead of venepunctures.^8,9^ In addition, the use of fingerpick sampling at home can potentially be cost-effective, as it will prevent extra visits to clinics and contributes to personalized natalizumab treatment.^3,5^ The objective of this study was to investigate whether a fingerprick can be used to measure natalizumab concentration in capillary blood samples in patients with RRMS treated with intravenous (IV) natalizumab.

## Methods

In this cross-sectional study, performed at the MS Center Amsterdam in The Netherlands, participants were recruited from the ongoing NEXT-MS trial (Clinicaltrials.gov NCT04225312). In the NEXT-MS trial participants receive personalized extended interval dosing of natalizumab based on natalizumab drug trough concentrations. Adult patients with RRMS are included (non-randomized) in three groups: standard interval dosing (SID) of every 4 weeks, personalized extended interval dosing (EID) with an aim natalizumab trough concentration of 10 μg/mL (EID10), and a subgroup with a lower aim of 5 µg/mL (EID5). Participants of the current study were selected consecutively when participants of the NEXT-MS trial visited the hospital for natalizumab treatment.

Capillary and venous blood samples were obtained prior to IV administration of natalizumab. First, capillary blood was obtained with the use of a fingerprick (contact-activated lancet, BD, Microtainer 2.0 by 1.5 mm) performed by a healthcare professional. Approximately 6–8 blood drops were obtained per sample, and were collected in capillary serum tubes (Minicollect 450533, Greiner). Next, venous blood (8 mL) was obtained in serum tubes through the cannula inserted for natalizumab administration. Samples were analyzed by Sanquin Diagnostic Services in Amsterdam, The Netherlands. Samples were centrifuged (1000*xg*) and serum was separated and stored at 4ºC until analyses were performed. Natalizumab concentrations were measured with a validated enzyme-linked immunosorbent assay in the same dilution as in routine diagnostics.^10^ Serum natalizumab concentrations between capillary and venous blood were compared using a Bland-Altman analysis. Spearman correlation coefficients were calculated to study the correlation of natalizumab serum concentration derived from capillary or venous blood. Participants provided written informed consent for measurement of natalizumab drug concentrations as part of the NEXT-MS trial (Medical Ethics Committee of Amsterdam UMC, location VUmc, registration numbers 2016.554 and 2019.552). Anonymized data will be shared upon reasonable request from any qualified investigator.

## Results

**Figure 1.**
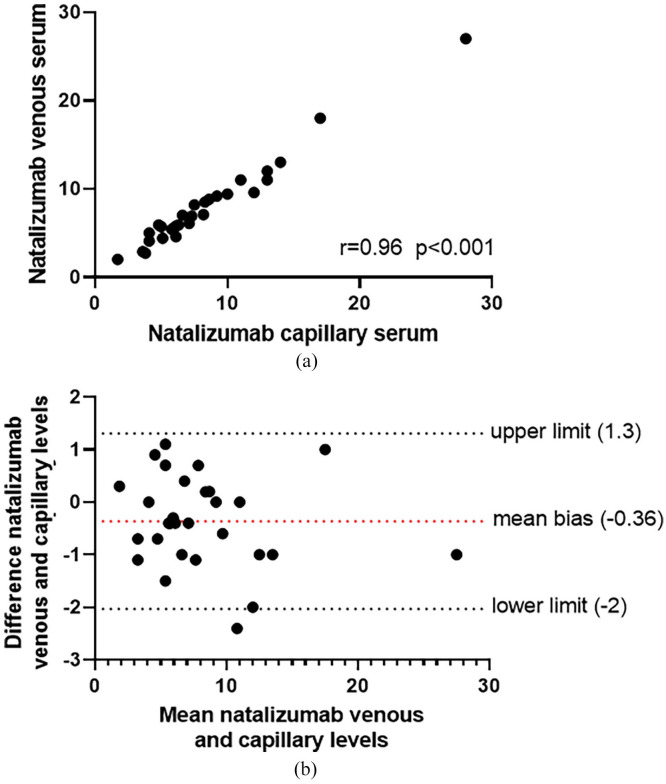
Capillary versus venous blood samples for measurement of serum natalizumab concentrations. (a) Scatterplot of natalizumab concentrations (µg/mL) for capillary (x-axis) versus venous (y-axis) blood samples as analyzed with a Spearman correlation. (b) Bland-Altman plot showing the mean bias and upper- and lower limit of the 95% agreement range of natalizumab concentrations in µg/mL.

Capillary and venous blood samples of 30 participants were obtained to compare serum natalizumab drug concentrations all participants were included in the personalized dosing study groups of the NEXT-MS trial. Half of the participants were on a 6 weeks treatment interval of natalizumab prior to the measurements (Table 1). Median natalizumab concentration of capillary blood was 6.9 µg/mL (interquartile range (IQR) 5.1–10.3 µg/mL). Median natalizumab concentration of venous blood was 6.5 µg/mL (IQR 5.3–9.5 µg/mL). There was a significant correlation (*r* = 0.96, *p* < 0.001) between natalizumab concentration from capillary and venous blood (Figure 1(a)). The mean difference between both methods was −0.36 µg/mL, calculated with a Bland-Altman analysis (Figure 1(b)). The limits of agreement were between −2 and 1.3 µg/mL.

**Table 1.** Patient characteristics.

	*N* = 30
Age, years	42.1 ± 12.6
Sex, % female	23 (77)
Body weight, kilograms	76.8 ± 12.8
Body mass index, kg/m^2^	25.5 ± 4.2
Disease duration, years	15.0 (7.0 to 21.5)
Duration of natalizumab treatment, years	8.5 (3.6 to 12.3)
Personalized dosing group (NEXT-MS trial)	
EID 5 group	21 (70)
EID 10 group	9 (30)
Natalizumab treatment interval	
4 weeks	5 (16.7)
5 weeks	2 (6.7)
6 weeks	15 (50.0)
7 weeks	5 (16.7)
8 weeks	2 (6.7)
9 weeks	1 (3.3)

Values are presented as means with standard deviation, medians with interquartile range, or frequencies with percentages. Disease duration and duration of natalizumab treatment were calculated until sample collection. All participants were included in the personalized dosing study groups of the ‘Personalized Extended Interval Dosing of Natalizumab in Relapsing Remitting Multiple Sclerosis (NEXT-MS)’ trial (EID5 with an aim drug trough concentration of 5 µg/mL and EID 10 with an aim drug trough concentration of 10 µg/mL). Participants on the 4 weeks natalizumab treatment interval (16.7%) already had drug levels in the targeted range (4/5) or had a baseline measurement before extending the treatment interval (1/5). EID = extended interval dosing.

## Discussion

In this study, we compared natalizumab concentration derived from capillary and venous blood in patients with RRMS treated with IV natalizumab. We show that a fingerprick is a reliable method to measure natalizumab drug concentrations, as the Bland-Altman plot showed no statistical difference between capillary and venous blood with a clinical acceptable upper and lower limit. The use of capillary blood enables physicians to monitor natalizumab drug concentrations without additional venepunctures, which will be particularly interesting for patients on SC natalizumab. In our cohort, natalizumab drug concentrations were relatively low, as the majority of patients were on personalized extended interval dosing based on natalizumab drug concentrations. However, we expect that patients will be treated with extended interval dosing more frequently in the future to reduce the risk of PML.^1,2^ Natalizumab SC is currently registered for administration within a healthcare environment, but has the potential to be administered at home in the future. As capillary blood can be collected by patients at home as well, remote monitoring of natalizumab drug concentrations can be just a fingerprick away.
